# Genome-Wide Association Study (GWAS) on Reproductive Seasonality in Indigenous Greek Sheep Breeds: Insights into Genetic Integrity

**DOI:** 10.3390/cimb47040279

**Published:** 2025-04-16

**Authors:** Danai Antonopoulou, George Symeon, Konstantinos Zaralis, Meni Avdi, Ilias S. Frydas, Ioannis A. Giantsis

**Affiliations:** 1 Department of Agriculture, Faculty of Agricultural Sciences, University of Western Macedonia, 53100 Florina, Greece; dsantono@agro.auth.gr (D.A.); kzaralis@uowm.gr (K.Z.); 2 Research Institute of Animal Science, HAO-Demeter, 58100 Giannitsa, Greece; gsymeon@elgo.gr; 3 Department of Animal Science, Faculty of Agriculture, Forestry and Natural Environment, Aristotle University of Thessaloniki, 54621 Thessaloniki, Greece; avdimel@agro.auth.gr; 4 Laboratory of Environmental Engineering, Department of Chemical Engineering, School of Engineering, Aristotle University of Thessaloniki, 54621 Thessaloniki, Greece; 5 HERACLES Research Center on the Exposome and Health, Center for Interdisciplinary Research and Innovation, Balkan Center, Bldg. B, 10th km Thessaloniki-Thermi Road, 57001 Thessaloniki, Greece

**Keywords:** small ruminants, seasonality, heterozygosity, genetic structure, autochthonous

## Abstract

A key feature in sheep biology is reproduction seasonality which concerns the cyclical occurrence of natural breeding, which therefore does not take place throughout the year. Since sheep are short-day breeders, the amount of daylight has an impact on their reproductive activity. The melatonin receptor subtype 1A (MTNR1A) gene is the primary gene that has been linked with seasonality. Nonetheless, information regarding the potential genetic association between other loci and the seasonality of sheep reproduction is scarce. Genome-wide association study (GWAS) is considered a cutting-edge methodology for comprehending the genetic architecture of complex traits since it enables the discovery of many markers linked to different features. In the present study, three indigenous Greek sheep breeds were investigated using GWAS—two of which presented strict patterns of reproduction seasonality, i.e., the Florina and Karagkouniko breeds, while the third one, i.e., the Chios breed had the ability to exhibit estrus throughout the year—in an attempt to detect the genetic loci linked with reproduction seasonality. All three breeds of investigated animals were purebred with Chios and Florina breeds originating from the Greek national stationary stock, whereas Karagkouniko originated from a commercial farm. Interestingly, a significant genetic differentiation of the national stationary stock groups was suggested by principal component analysis, phylogenetic analysis, and admixture and spatial point patterns, with these two breeds being less heterogeneous. This finding highlights the value of stationary stocks towards the maintenance of genetic integrity in indigenous sheep, demonstrating the Greek station’s critical role in the conservation of native sheep breeds. On the other hand, according to the GWAS data analysis, no genetic loci were correlated with reproduction seasonality, emphasizing the MTNR1A gene as the main determinant of the seasonality in native non genetically improved breeds.

## 1. Introduction

Sheep farming is a traditional yet important agricultural activity in Greece, with a long history dating back to ancient times. It represents an essential part of the primary sector of production in Greece, particularly in rural, marginal, and mountainous regions where the terrain is less suitable for arable production or other forms of farming. Dairy production in Greece relies mainly on sheep, with goats playing an important but secondary role, whereas dairy sheep production ranks first in Europe producing nearly 950,000 tonnes of milk, positioning Greece as the leading producer in the European Union [1]. Good reproduction management of the flocks plays a crucial role in maintaining the sustainability and productivity of the sector in addition to ensuring the economic viability of sheep farmers. Depending on the seasonality of the breeds and genotypes, the breeding season typically starts early in autumn and can continue up to early March while anestrus periods occur during spring and summer [2,3,4]. During this period, flocks are carefully managed by farmers to ensure successful mating and optimal breeding outcomes.

In general, reproduction seasonality in sheep refers to the natural breeding patterns and production cycles that occur at different times of the year. Sheep are short-day breeders, which means that their reproductive activity is influenced by the length of daylight. Most sheep breeds are polyestrous, meaning they have multiple estrous cycles within a single breeding season. In this respect, seasonality plays a crucial role in determining the optimal times of the flock for mating, which in turn can influence the lambing time, health and welfare management, the productivity of the flock, and the availability of milk in the market and lambs for fattening. It should be noted that seasonality has been proposed to exhibit a strong genetic background [5,6].

A primary mechanism underlying seasonal reproduction is a significant increase in the responsiveness of gonadotropin-releasing hormone (GnRH) neurons to the negative feedback effects of estradiol, which peaks during anestrous and declines during the mating season. This intrinsic physiological clock system is essential for the temporal adaptations of animals. Daily exposure to light and other stimuli consistently recalibrates this clock and aligns it with climatic conditions. The clock output channels subsequently regulate different physiological features. A primary output from the circadian clock is the nocturnal release of the hormone melatonin, which is produced and secreted in vertebrates by the pineal gland [7,8].

Melatonin does not directly cause seasonal changes in reproduction but it rather seems to synchronize an endogenous circannual rhythm [7]. It is currently established that melatonin is synthesized by various peripheral tissues and organs, including the retina, Harderian gland, ovary, testis, bone marrow, lymphocytes, hepatic cholangiocytes, gastrointestinal tract, and skin [9]. Notably, it was revealed that the skin and stomach synthesize higher quantities of melatonin than the pineal gland [10]. Consequently, the notion of melatonin as a neurohormone was revised, elucidating the widespread occurrence of extra-pineal melatonin in animals [11]. A neurochemical network comprising the retina, the suprachiasmatic nucleus, and the pineal gland governs circadian rhythms that align with changes in daylight photoperiod. It has also been proposed that rams in many breeds exhibit reproductive seasonality in testosterone secretion, sperm quantity, and quality [12]. In this context, when the ewe’s ovulation and estrus are arrested, the ram’s spermatogenesis and sexual activity continue unabated. In general, all of these aspects are highly exhibited toward the end of summer and autumn, and are low at the end of winter and spring [13]. The length of the nightly increase in melatonin in the pineal gland due to photoperiod and the sensitivity of target organs are likely crucial elements in triggering the endocrine alterations that result in breeding activity in seasonal breeds [14]. Melatonin’s lipophilic nature allows it to cross all cell membranes and enter all tissues, but once in the system, it is notably concentrated in the ovaries [15]. Furthermore, melatonin influences the pulse of gonadotropin-releasing hormone (GnRH) in the hypothalamus via melatonin receptor type 1 (MT1) [16]. Thus, the main gene proposed to be associated with the seasonality of sheep is the melatonin receptor subtype 1A (MNTR1A) gene [5,6].

It should be noted that so far, apart from the MNTR1A gene, no other gene has been strictly correlated with reproduction seasonality in sheep, with only a few exceptions of SNPs and genetic loci, such as the growth and differentiation factor 9 (GDF9), the bone morphogenetic protein 15 (BMP15), and the Lysine demethylase 4B (KDM4B), as reviewed in [17], however, a direct correlation has not been confirmed. Nevertheless, the whole genome of sheep has been intensively analyzed and characterized. Thus, to the best of our knowledge, except for MNTR1A, there is a lack of genetic insights into whether other loci are related to seasonality in sheep reproduction. In this context, genome-wide association studies (GWASs) allow the detection of multiple markers associated with various features and are considered a state-of-the-art approach to understanding the genetic architecture of complex traits [18], such as seasonality in reproduction.

To the best of our knowledge, no other loci have been confirmed to influence reproductive seasonality in sheep. In this respect, the purpose of the current study was to uncover novel SNPs, genes, or non-coding genetic loci that may be associated with seasonality in sheep reproduction. To achieve this goal, breeds that are known to differ in their seasonality in reproduction represent an ideal model. Hence, three Greek indigenous breeds were selected, which are characterized by different levels of reproductive seasonality. More specifically, single nucleotide polymorphisms (SNPs) that have been shown to have relationships with reproductive parameters were investigated in the three indigenous Greek sheep breeds, i.e., Chios, Florina, and Karagouniko, two of which (Florina and Karagkouniko) are characterized by seasonality in reproduction, whereas the Chios breed exhibits estrus during longer periods [3]. Additionally, the genetic relationships of these breeds were investigated by the means of SNPs. It must be emphasized that the two of them constitute the formal stationary stock regulated by the Greek government and are bred in the Research Institute of Animal Science under the constant supervision of the research personnel.

## 2. Materials and Methods

### 2.1. Samples and Molecular Analyses

The study involved a total of 96 ewes, all of which were between 2 and 3 years old. Out of these animals, 30 belonged to the Chios breed, another 30 animals belonged to the Florina breed, and 36 to the Karagkouniko breed. The Chios and Florina breeds are kept and bred at the Research Institute of Animal Science of ELGO-DEMETER in Paralimni Giannitson while the Karagkouniko animals originated from a commercial farm in Karditsa, Region of Thessaly, Greece. The Karagkouniko and Florina sheep breeds originate from the mainland of Greece, whereas the Chios sheep breed originates from the Greek island of Chios. The Chios breed is a non-seasonal reproduction breed, whereas the remaining two exhibit a strong seasonal reproduction behavior and a long anestrus period.

Blood samples were collected from the jugular vein of each ewe using venoject needles into 10 mL vacutainer tubes containing EDTA or sodium heparin (BD Vacutainer Systems, Rutherford, NJ, USA) and were stored at 4 °C until further analysis. DNA was extracted from 150 μL of the collected blood utilizing the PureLink Genomic DNA Mini Kit (Invitrogen, Waltham, MA, USA) according to the manufacturer’s recommended protocol. The quality and quantity of the extracted DNAs were evaluated by analyzing the 260/280 and 260/230 ratios in a Q5000 UV–Vis Spectrophotometer (Quawell, San Jose, CA, USA). All the animal manipulations were performed taking into careful consideration and following the EU Directive guidelines concerning the protection of animals’ usage for scientific purposes (2010/63/EU). Since the sample collection did not include any endangered or protected species, nor animals were injured or sacrificed, no more specific permits were required for the implementation of the study.

### 2.2. Genotyping and Bioinformatic Analysis—Data and Quality Control

All the animals were genotyped using the Illumina Ovine 50 K SNP BeadChip array and the GenomeStudio software v.2.0.5 (Illumina Inc, San Diego, CA, USA). Genotypes with a GenCall score greater than 0.3 were considered valid for further analysis. In total, 50 k genotypes of 96 sheep samples from mainland and island Greece were used. More specifically, 66 samples originate from the mainland (Florina, n = 30 and Karagkouniko, n = 36), and 30 sheep genotypes originate from island Greece (Chios, n = 30). Quality control (QC) was performed at a sample and marker level. All the unmapped SNPs, those mapping to sexual chromosomes, SNPs with a genotyping rate lower than 90%, or those below a minor allele frequency threshold of 0.05 were removed. To avoid deviations from the Hardy–Weinberg equilibrium due to genotyping errors, the SNPs that did not pass the Hardy–Weinberg test for *p* ≤ 0.001 were removed as well. A total of 38,893 SNPs located on the 26 ovine autosomes passed the quality control for the whole sample analyzed.

At the animal level, 5 samples were excluded from analyses due to a call rate lower than 0.90. An additional number of 36 related animals were removed based on identity by descent (IBD) degree of recent shared ancestry (*p*^) threshold of 0.25 (second-degree relatives). At the marker level, 18,331 of the 62,329 original number of SNPs were excluded due to a call rate lower than 0.95, minor allele frequency (MAF) lower than 0.05, deviation from Hardy–Weinberg equilibrium (HWE) using a Fisher exact test *p*-value < 10^−5^, and linkage disequilibrium (LD) r^2^ levels (r^2^ > 0.50, window size: 50 SNPs, increment: 5 SNPs) and autosomal mapped SNPs. The application of QC criteria at the marker level resulted in a total number of 43,998 autosomal mapped SNPs retained for further analyses.

### 2.3. Population Structure, Heterozygosity, and Phylogeny Analyses

All the computations to assess the intra-individual distance matrix (complimentary to the intra-individual identity-by-state matrix), and the between individuals’ relationships were summarized by implementing a principal component analysis (PCA) on such distance matrix and were performed using the software PLINK (v.1.9) in R (v.4.4.1.) [19]. The mean of heterozygosity was estimated by the F coefficient, whereas eigenvectors computed for each animal via PCA were used to construct dispersion plots. Finally, an unrooted phylogenetic tree showing pairwise Reynold’s genetic distances between the three different breeds was constructed by the R software (v.4.4.1) using the R packages ‘ape’ and ‘phanogorn’ [20,21].

### 2.4. Genome-Wide Association Analysis (GWAS)

Due to the small breed sample size, genome-wide association analysis (GWAS) was performed with and without the application of relatedness factor (IBD) and compared the two mainland breeds, i.e., Florina and Karagkouniko to the island breed of Chios. To detect significant associations between SNPs (fitted as fixed effect covariates), GWAS was performed as a single locus mixed (additive) model using the EMMAX algorithm [22], and the two origins were coded as binary dependent variables (0: island; 1: mainland), and no other covariate was included in the model as that would intervene with the dependent variable. During this analysis, animals’ relationships were included in the mixed model as random effects via the PCA. The genome-wide significance of SNPs was declared using an FDR *p*-value lower than 0.05. The expected (null) distribution of the statistic test was assessed by using Q-Q plots, and in addition to the genomic inflation factor (λ) it was feasible to estimate the extent of the potential systematic bias due to the analytical approach or to the breeding population [23]. All the above analyses were carried out in R (v.4.4.1) using PLINK v.1.9.

## 3. Results

### 3.1. Genetic Structure and Heterozygosity

**Figure 1:**
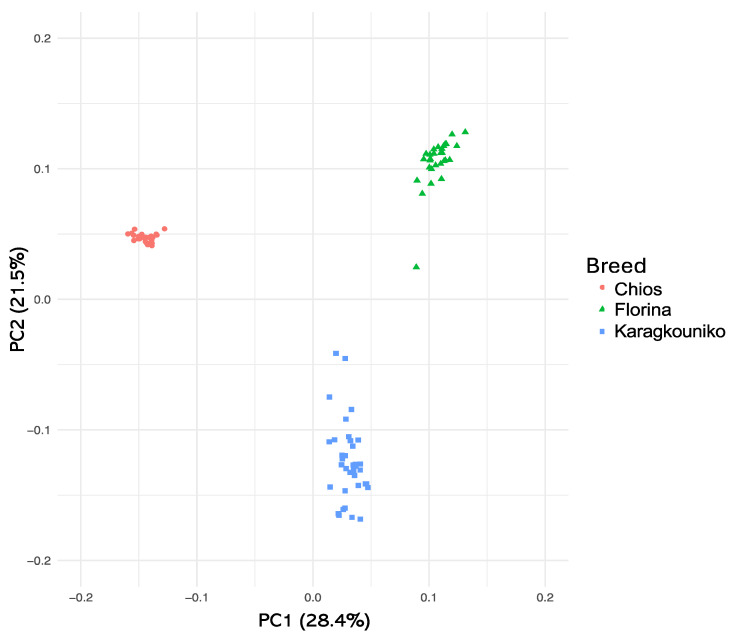
Two-dimensional plot of the first three PCs derived from PCA on the animals’ genomic relationships. A total number of three clusters were created, one with the genotypes from Chios, a second with the genotypes from Florina islands, and a third one with the genotypes from Karagouniko.

**Figure 2:**
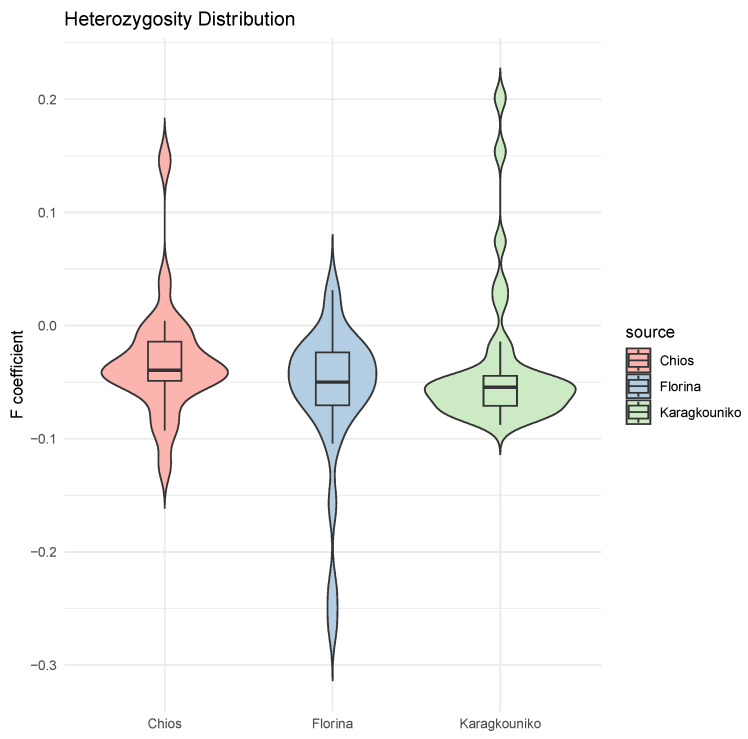
Mean of heterozygosity (F coefficient) distribution in the sheep breeds (Chios, Florina, and Karagkouniko). Individual values and standard error of the means are presented.

**Figure 3:**
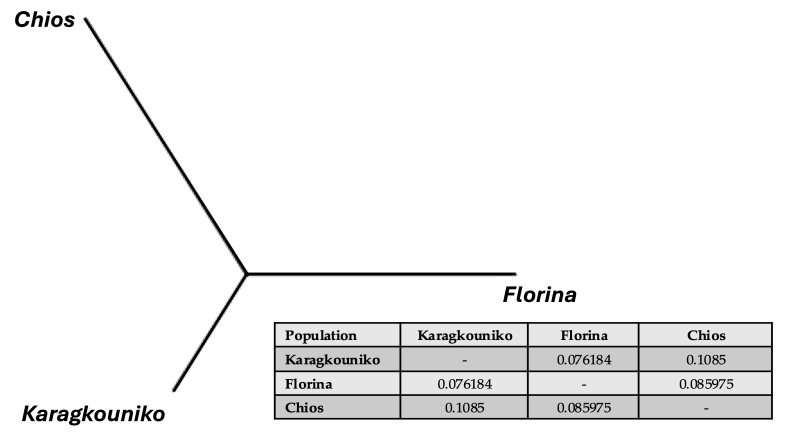
An unrooted phylogenetic tree showing the genetic Reynolds’ between-breed distances and the pairwise distance Fst values.

The genomic relationships between the three different sheep breeds are shown in PCA (Figure 1), where the two mainland sheep breeds (Florina and Karagkouniko) and the one island breed from Chios are depicted. The PCA revealed the formation of three different clusters. All three breeds are shown to be genetically distinguished from each other. The Karagkouniko breed (illustrated with blue dots) demonstrated the highest within-sample size variation in animals’ relationships as it reached 28.4%. The highest discriminatory power (DP) was observed in the Chios breed in view of the DP shown from the other two breeds, i.e., Florina and Karagkouniko. The principal components and phylogenetic analysis along with the admixture and spatial point pattern analyses suggested the genetic differentiation of ‘mainland-island’ populations. In line with these results, the heterozygosity analysis revealed the Chios breed to be the least heterogenous breed, followed by the second Greek stationary breed of Florina, whereas the Karagkouniko animals, originating from a commercial farm, exhibited the highest heterogeneity (Figure 2). In addition, the PCA results are confirmed by the unrooted generated phylogenetic tree in which the genetic discrimination of the two mainland (Florina and Karagkouniko) and the island branch genotype (Chios) is demonstrated, as well as high levels of genetic distance between the two mainland branch genotypes of Florina and Karagkouniko (Figure 3).

### 3.2. GWAS Analysis

**Figure 4:**
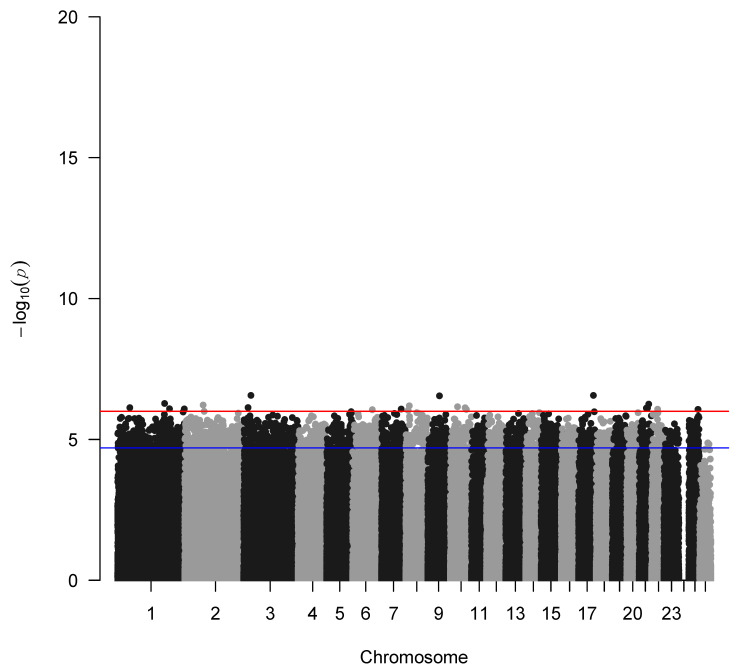
Manhattan plot of –log10 (*p*-values) of SNPs (top) across autosomes without applying the IBD factor. The red line denotes the threshold (−log10 (*p*-value) = 5.893) for genome-wide significance, and the blue line denotes suggestive significance.

**Figure 5:**
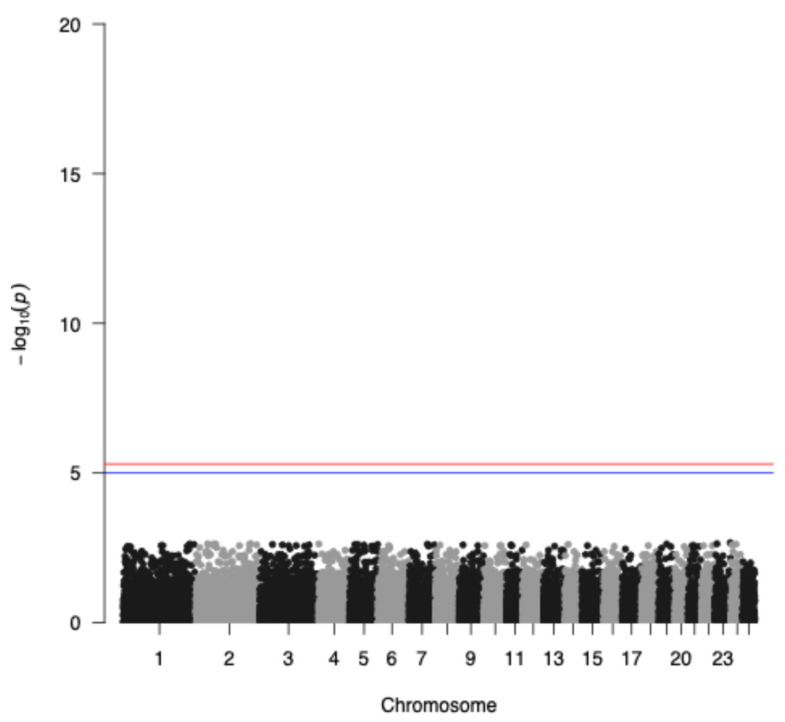
Manhattan plot applying the IBD factor of −log10 (*p*-values) of SNPs (top) across autosomes. The red line denotes the threshold (−log10 (*p*-value) = 5.893) for genome-wide significance, and the blue line denotes suggestive significance.

The genome-wide association analysis without the application of the IBD factor is depicted in Figure 4 indicating a genetic trend to associate a few SNPs with seasonality with the highest representative s71757.1 in chromosome 1, whereas when incorporating the IBD factor, as expected, no significant SNPs were identified due to the small sample size of the study populations (Figure 5). Additionally, GWAS analysis without applying the IBD factor showed 31 potential candidate SNPs (*p* < 0.05) (Table 1) proposing again an association trend.

## 4. Discussion

In this research, for the first time, the genetic background of three native Greek breeds has been explored in correlation with the seasonality of reproduction based on genome-wide association study (GWAS) analyses. Previous GWAS analyses of other indigenous Greek sheep breeds focused on local adaptation and relative resilience [24,25,26], demonstrating several associated SNPs with these traits. Nevertheless, none of these genetic loci was statistically significant here, whereas reproduction seasonality has not been studied so far in Eastern Mediterranean indigenous sheep breeds. To this end, based on the IBD factor analysis, neither the whole GWAS nor specifically the 93 SNPs previously linked with various reproductive traits were strictly correlated with seasonality in reproduction. Nevertheless, there was an important trend towards the association of several SNPs with seasonality, with a particular one (s71757.1) already correlated previously with reproductive traits. This SNP is located in the ST6GALNAC3 gene, which although mostly evinced to be implicated in biochemical pathways, i.e., sialyltransferases that transfer sialic acids, has also been associated with litter size in sheep [27]. Further, when comparing our results with the only available previous GWAS analysis that investigated SNP correlation with seasonality in reproduction in western Mediterranean sheep breeds [28], interestingly, none of the potentially associated SNPs was identical. Nevertheless, in line with these outcomes, one SNP at chromosome 8 revealed an association at the chromosome-wise level. Overall, however, despite the limited number of animals investigated, mainly attributed to the low population numbers of indigenous breeds in Greece, the current results indicate that there is no strict correlation between seasonality and any of the investigated SNPs with only a few exceptions that verified a clear genetic trend of seasonality of reproduction in sheep. These inferences indicate the complexity of this trait, which is, therefore, not directly dependent on reproduction-related hormones, but mainly on photoperiodism. Interestingly also, among the 93 SNPs investigated for the seasonality investigation study, none is located within the MTNR1A gene. These findings support the assumption that MTNR1A is the main gene that can influence reproductive seasonality in sheep. It can be stated that the molecular study of the MTNR1A gene can serve as an excellent methodology for marker-assisted selection, optimizing estrus synchronization, especially for seasonal sheep breeds [29]. Previously, it was proven that sheep possessing the CC/CC and CT/CT genotypes exhibit earlier reproductive recovery than those with the TT/TT genotypes [6]. Consequently, animals with genotypes CC/CC and CT/CT seem to be less influenced by variations in photoperiod, resulting in a shorter photo-refractoriness period compared to TT/TT animals [30]. More recently, the SNPs rs430181568, rs407388227, and rs403212791 of the MTNR1A gene were also correlated with variations in reproductive rates [31,32]. Additionally, other genes, such as the leptin gene [33] and the PAK1, CYP19A1, and PER1 genes [34], have been shown to potentially have an effect on reproduction seasonality, with these outcomes, however, being yet to be confirmed by larger populations and future studies, similarly with the potentially associated SNPs in our study.

Among these investigated genetic loci, several are located in genes previously associated with reproduction parameters [35]. The BMP15 gene is foremost among candidate genes regulating ovarian function, ovulation rate, and therefore, prolificacy in ovine species, with 10 independent mutations detected out of the 17 documented [36]. In addition to the FecB mutation at BMPR1B, a notable correlation between the missense mutation rs406686139 in the seasonal lambing-associated TSHR gene and litter size was identified [37]. The heightened diversity in litter size resulting from the FecL mutation is not detrimental, providing the case that farmers can accept triplets. Nevertheless, as several farmers aim to restrict the occurrence of triplets and prevent litter sizes above three, the management of the FecL gene should be linked to a decrease in litter size variability, potentially by canalized selection [38]. The putative genes linked to litter size in Pelibuey sheep that may play a role in reproduction include CLSTN2, MTMR2, CCDC174, NOM1, ANKRD11, DLG1, ALPK3, ROBO2, CGA, and KDM4A [39]. Growth differentiation factor 9 (GDF9), also known as the FecG gene, is a crucial autosomal significant gene that influences prolificacy and is vital for optimal folliculogenesis. This gene encodes a member of the transforming growth factor-beta superfamily. The GDF9 S395F and S427R mutant variants enhance the ovulation rate in heterozygotes, however, the homozygous form results in infertility [40]. A correlation was found between the mutations in the GDF9 and BMP15 genes in women with dizygotic twins, along with the clinical importance of these oocyte variables in the development of primary ovarian insufficiency [41]. Thirty-five QTL areas identified on sixteen OARs exhibited significant associations with Merino ram semen volume, gross motility, concentration, and percentage of post-thaw motility and several genes, including SORD, SH2B1, and NT5E, essential for spermatogenesis, sperm motility, and enhanced motility post-cryopreservation. Also, genes such as PADI2, RAB3B, and ALDOA, previously linked to spermatozoal maturation, the acrosome reaction, and successful conception, were also found [42]. Numerous polymorphisms in DGAT1 have been recorded across various animal species, including cattle, buffalo, goats, and sheep, in relation to milk production features. Furthermore, DGAT1 has been examined for its influence on meat production features in cattle, sheep, and goats [43]. An analysis of the coding sequences of the FABP4 gene, which is located between genes associated with reproductive traits, produced results that corroborated the substantial influence of the detected p.61Thr > Asp SNP on growth characteristic metrics in the Awassi and Karakul breeds [44]. In the EPHA6 (ephrin type-A receptor 6) gene, a definitive connection was observed between the SNP—OAR1_172690647.1, located on chromosome 1 (160,114,886 bp, rs402032081), and fertility traits in Polish Mountain Sheep [45]. A single nucleotide polymorphism (SNP) identified as detrimental, situated in the extracellular domain of the LEPR gene (snp_ex8), exhibited a substantial correlation with the estrous cycling months in Rasa Aragonesa sheep, affirming the involvement of the LEPR gene in reproductive seasonality among ruminants [33]. The discovered SNPs were distributed across multiple chromosomes, indicating the distinct genetic pathways contributing to prolificacy variation in each breed. The several layers constituting the follicles necessitate communication and the transport of hormones and nutrients to the oocyte, hence underscoring the importance of the pathways associated with cell signaling and communication [46].

Sheep farming is probably the most important sector of livestock production in several Mediterranean countries, being of considerable value due to its impact on the rural economy and due to its cultural and environmental significance. In temperate latitudes, such as Greece, animals contend with periodic seasonal variations in temperature and food supply. Sheep in temperate regions have seasonal breeding behavior. Among the seasonal signals, photoperiod is the most dependable indicator, utilized by animals to align their internal annual rhythms of reproduction and physiology with the time of year [47]. Particularly, indigenous Greek sheep breeds are seen as being of great significance for the local economy in terms of both conservation and regional development; yet, they frequently have lengthy anestrus periods, which results in limited production of dairy products during certain times of the year [2]. Two of these breeds are strictly seasonal in terms of reproduction, namely Florina and the Karagkouniko [29]. The Chios breed is non-seasonal and has a long breeding period throughout the year [3]. Thus, sheep bred in temperate regions often exhibit seasonal reproductive behavior. The reproductive season typically commences in autumn and concludes in winter, with anestrus occurring in spring and summer. An intrinsic circannual rhythm, influenced and coordinated by the annual photoperiod cycle, governs the initiation and conclusion of the breeding season [15]. Estrus activity and ovulation rate in farm animals are known to be highly influenced by body fat reserves with animals of very low (i.e., <2.5) or very high (i.e., >3.5) body condition score (BCS) exhibiting reduced estrogenic activity, inability to reactivate the ovulatory activity effectively, and low conception rates [48,49].

Most importantly, however, seasonality depends on the genetic synthesis of the breed. Among the breeds examined in the present study, Chios sheep have ovulatory activity throughout the year except for a short period of time for about half the population [2]. From September till the conclusion of winter, ewes display consistent cyclicity. The behavior of the ewes is analogous throughout the spring season, during which some animals halt cycling, while others maintain normal cyclicity [50]. Contrariwise, the Karagkouniko and Florina breeds exhibit estrus in a pronounced seasonal rhythm, specifically from mid-summer to late fall [51]. In rams, photoperiod appears to significantly influence the testosterone levels of the Karagkouniko and Chios breeds in comparison to other environmental factors such as temperature [52]. Chios sheep had markedly elevated testosterone levels compared to the Karagkouniko [51].

Concerning the population genetic structure results, the present study indicates the genetic integrity of the animals originating from the Greek purebred public breeding station. On the contrary, in line with a previous study based on SNPs as well, which demonstrated high levels of genetic heterogeneity in some breeds including Chios [53], sheep from the commercial farm are more heterogeneous (Figure 2), putting at risk the conservation genetic status of the commercially farmed breeds. This phenomenon is probably a result of the high gene flow rates among breeds, attributed to the continuous admixture evinced by various molecular markers such as microsatellites [54] and mtDNA and nuclear gene sequencing [55]. In the public purebred station, detailed breeding data are kept for over twenty years, and the animals are under the constant supervision of animal scientists. The breeding scheme is carefully designed on the one hand to avoid in-breeding and on the other hand to promote favorable characteristics like disease resistance and milk production. Indeed, a primary issue in sheep farming is unregulated cross-breeding, when breeding occurs outside the established selection strategies, with farmers favoring more productive indigenous or imported breeds due to mounting economic pressures. These activities result in genetic dilution, wherein an excessive prevalence of non-local genes diminishes the relative frequency of native genes within the population. They promote genetic homogeneity through the elimination of rare or specific variants, disrupting co-adapted gene complexes, which threaten the integrity and viability of local populations [56]. The PCA plot results suggest substantial genetic structuring among the three sheep breeds examined: Florina, Karagkouniko, and Chios. The Florina and Chios sheep, bred at the Research Institute of Animal Science of ELGO-DEMETER, exhibit considerable genetic variation, exceeding the Karagkouniko sheep cultivated at a commercial farm. The interpopulation genetic homogeneity in the two purebred breeds is of very high importance for future breeding programs. Indigenous breeds from Greece like Florina are generally characterized by low productivity, but at the same time, high tolerance in tough weather conditions and oligotrophic pastures. Based on their genetic integrity, as revealed by GWAs, they may constitute excellent units for selective and cross-breeding to improve the adaptation of improved breeds.

Similarly, in the UK, native breed research conducted on animals from commercial farms showed minimal genetic differentiation, possessing the lowest kinship coefficients. These findings were expected as these breeds are extensively utilized in commercial sheep production in the UK [57]. In a recent study on two Czech sheep breeds, also from commercial farms, a wide range of in-breeding (FHOM) among individuals was exhibited in both breeds from 0.04 to 0.16 in Sumava sheep and from 0.13 to 0.12 in Wallachian sheep [58]. In a study on the Pantaneiro sheep and Texel breed of Brazil, the PCA plot indicated that the Pantaneiro breed demonstrated superior genetic variety, but Texel, characterized by a more regulated population, exhibited anticipated traits of elevated in-breeding and diminished genetic interchange [59]. On the contrary, a study on Hu sheep that were bred in Lanzhou Xinyuan Modern Agriculture Technology Development in China exhibited high genetic diversity among other breeds [60]. In 2020, in a study on 24 sheep breeds bred in Ireland, where most of the animals were recorded to be purebred, Suffolk and Border Leicester were the breeds that were genetically most diverse from each other, while the other breeds integrated clusters in the PCA and exhibited the lowest pairwise Fst [61].

This suggests that sheep breeds raised in a controlled environment, such as a research facility, demonstrate more genetic homogeneity, as observed in the Florina and Chios sheep in this study. Additionally, despite the genetic admixture that has been reported in indigenous breeds [55], the present findings demonstrate that with proper management strategies applied, the distinct genetic profile of indigenous breeds can be maintained.

## 5. Conclusions

Based on the present study, the genetic background of reproduction seasonality is clearly confirmed. Despite the great role of the MTNR1A gene being the primary factor that determines the seasonality of Greek sheep breeds, several other SNPs were potentially associated with this complex trait. The location of these genetic loci, in line with previous studies, demonstrates the high complexity of seasonality of reproduction in sheep, influenced by various physiological functions. More importantly, from a marker-assisted selection point of view towards genetic improvement for seasonality in the reproduction reduction in sheep, it is inferred that different genetic loci may enact different roles in breeds from different regions. Additionally, our findings indicated the important role of the Greek station in the conservation and genetic integrity of indigenous sheep breeds.

## Figures and Tables

**Table 1:** Potentially associated candidate SNPs with seasonality in reproduction in three investigated breeds.

CHR	SNP	UNADJ	GC	BONF	HOLM	SIDAK_SS	SIDAK_SD	FDR_BH	FDR_BY
17	OAR17_66137286.1	2.713 × 10^−7^	0.08945	0.01157	0.01157	0.0115	0.0115	0.0003336	0.003749
3	OAR3_32230041.1	2.714 × 10^−7^	0.08946	0.01157	0.01157	0.01151	0.01151	0.0003336	0.003749
9	s28425.1	2.816 × 10^−7^	0.08989	0.01201	0.01201	0.01194	0.01194	0.0003336	0.003749
1	OAR1_209228482.1	5.24 × 10^−7^	0.09752	0.02235	0.02235	0.0221	0.0221	0.0003336	0.003749
21	ilmnseq_rs604725717	5.577 × 10^−7^	0.09832	0.02378	0.02378	0.0235	0.0235	0.0003336	0.003749
2	OAR2_81887812.1	6.002 × 10^−7^	0.09928	0.0256	0.0256	0.02527	0.02527	0.0003336	0.003749
8	OAR8_15349408.1	6.368 × 10^−7^	0.1001	0.02716	0.02716	0.02679	0.02679	0.0003336	0.003749
10	OAR10_31896326.1	6.886 × 10^−7^	0.1011	0.02937	0.02936	0.02894	0.02894	0.0003336	0.003749
3	OAR3_19421671.1	7.366 × 10^−7^	0.102	0.03142	0.03141	0.03093	0.03092	0.0003336	0.003749
1	s71757.1	7.432 × 10^−7^	0.1021	0.0317	0.03169	0.0312	0.03119	0.0003336	0.003749
10	OAR10_65844667.1	7.467 × 10^−7^	0.1022	0.03185	0.03184	0.03134	0.03134	0.0003336	0.003749
21	s35322.1	7.641 × 10^−7^	0.1025	0.03259	0.03258	0.03206	0.03206	0.0003336	0.003749
21	s56586.1	7.674 × 10^−7^	0.1026	0.03273	0.03272	0.0322	0.03219	0.0003336	0.003749
1	OAR1_231434082.1	8.13 × 10^−7^	0.1033	0.03467	0.03466	0.03408	0.03407	0.0003336	0.003749
1	OAR1_297285976.1	8.223 × 10^−7^	0.1035	0.03507	0.03506	0.03446	0.03445	0.0003336	0.003749
7	s42963.1	8.295 × 10^−7^	0.1036	0.03538	0.03537	0.03476	0.03475	0.0003336	0.003749
22	s27062.1	8.411 × 10^−7^	0.1038	0.03587	0.03586	0.03524	0.03522	0.0003336	0.003749
25	s37856.1	8.502 × 10^−7^	0.1039	0.03626	0.03625	0.03561	0.0356	0.0003336	0.003749
10	OAR10_71264685.1	8.614 × 10^−7^	0.1041	0.03674	0.03672	0.03607	0.03606	0.0003336	0.003749
6	OAR6_87983645.1	8.775 × 10^−7^	0.1044	0.03743	0.03741	0.03674	0.03672	0.0003336	0.003749
8	OAR8_10222234.1	9.306 × 10^−7^	0.1052	0.03969	0.03967	0.03891	0.0389	0.0003336	0.003749
2	OAR2_86483053.1	1.007 × 10^−6^	0.1063	0.04294	0.04292	0.04203	0.04201	0.0003336	0.003749
5	OAR5_109887031_X.1	1.026 × 10^−6^	0.1066	0.04376	0.04374	0.04282	0.04279	0.0003336	0.003749
17	ilmnseq_rs424436982	1.037 × 10^−6^	0.1067	0.04423	0.04421	0.04327	0.04325	0.0003336	0.003749
1	OAR1_292286259.1	1.056 × 10^−6^	0.107	0.04502	0.04499	0.04402	0.04399	0.0003336	0.003749
14	ilmnseq_rs428524353	1.114 × 10^−6^	0.1077	0.04752	0.04749	0.0464	0.04638	0.0003336	0.003749
8	OAR8_48743831.1	1.117 × 10^−6^	0.1078	0.04763	0.0476	0.04652	0.04649	0.0003336	0.003749
20	OAR20_50272411.1	1.12 × 10^−6^	0.1078	0.04777	0.04774	0.04665	0.04662	0.0003336	0.003749
22	OAR22_28251375.1	1.136 × 10^−6^	0.108	0.04847	0.04844	0.04731	0.04728	0.0003336	0.003749
2	OAR2_238706074.1	1.155 × 10^−6^	0.1083	0.04928	0.04924	0.04808	0.04805	0.0003336	0.003749
5	OAR5_110584928.1	1.16 × 10^−6^	0.1083	0.04948	0.04944	0.04828	0.04824	0.0003336	0.003749
13	s63375.1	1.181 × 10^−6^	0.1086	0.05037	0.05033	0.04912	0.04909	0.0003336	0.003749

## Data Availability

Data are included within the manuscript. Further details are available upon request.
